# Advances in Molecular Imaging and Radionuclide Therapy of Neuroendocrine Tumors

**DOI:** 10.3390/jcm9113679

**Published:** 2020-11-16

**Authors:** Anna Yordanova, Hans-Jürgen Biersack, Hojjat Ahmadzadehfar

**Affiliations:** 1 Department of Radiology, St. Marien Hospital Bonn, 53115 Bonn, Germany; anna.yordanova@mail.com; 2 Department of Nuclear Medicine, University Hospital Bonn, 53127 Bonn, Germany; 3 Department of Nuclear Medicine, Klinikum Westfalen, 44309 Dortmund, Germany

**Keywords:** molecular imaging, PET, radionuclide therapy, PRRT, neuroendocrine tumors, NET

## Abstract

Neuroendocrine neoplasms make up a heterogeneous group of tumors with inter-patient and intra-patient variabilities. Molecular imaging can help to identify and characterize neuroendocrine tumors (NETs). Furthermore, imaging and treatment with novel theranostics agents offers a new, tailored approach to managing NETs. Recent advances in the management of NETs aim to enhance the effectiveness of targeted treatment with either modifications of known substances or the development of new substances with better targeting features. There have been several attempts to increase the detectability of NET lesions via positron emission tomography (PET) imaging and improvements in pretreatment planning using dosimetry. Especially notable is PET imaging with the radionuclide Copper-64. Increasing interest is also being paid to theranostics of grade 3 and purely differentiated NETs, for example, via targeting of the C-X-C motif chemokine receptor 4 (CXCR4). The aim of this review is to summarize the most relevant recent studies, which present promising new agents in molecular imaging and therapy for NETs, novel combination therapies and new applications of existing molecular imaging modalities in nuclear medicine.

## 1. Introduction: Current Knowledge and Aims of this Review

In modern oncology, imaging is necessary for treatment planning, tumor staging and follow-up. Molecular imaging with positron emission tomography (PET) or single photon emission computed tomography (SPECT) enables real-time visualization of tumor receptors, metabolism and pathogenesis. PET exhibits better image quality and sensitivity over SPECT. For that reason, current theranostic approaches prefer PET, and thus PET peptide tracers will be the focus of this review. PET imaging can be used to select the most suitable candidates for targeted treatment and provides early evidence of treatment response. At the same time, treatment with highly targeted radiopharmaceuticals is associated with good tolerability, which is important for the maintenance of quality of life [1,2,3].

Neuroendocrine tumors (NETs) can originate from any part of the body, most commonly from the gastrointestinal tract and the lungs. The cells of NETs compound characteristics of nerve cells and endocrine cells [4,5,6]. Well-differentiated NETs overexpress somatostatin receptors (SSTRs), especially sub-type SSTR-2. The targeting of SSTRs with theranostic agents in NETs has been available since 1989 and has paved the way for modern theranostics, which use different radiolabeling of the same agent or very similar agents for treatment and imaging to select patients who are likely to respond to the treatment [7,8,9,10].

In PET diagnostics, there are three routine tracers labelled with Gallium-68, which specifically bind SSTRs (sensitivity up to 97% and specificity up to 92%): DOTA-TOC, DOTA-TATE and DOTA-NOC [11,12,13,14,15]. SSTR-imaging can be used for diagnosing NETs and for selecting patients for peptide receptor radionuclide therapy (PRRT). In the past few years, PRRT has gained increasing attention since the randomized prospective phase III trial NETTER-1 showed, in patients with midgut NET, clear clinical benefits from PRRT with [^177^Lu]Lu-DOTA-TATE compared to the somatostatin receptor analog Sandostatin^®^ LAR: progression-free survival (PFS) with the median not reached at the time of the primary endpoint analysis vs. 8.4 months (*p* < 0.001), better overall response (18% vs. 3%) and a presumably longer overall survival (OS) (median not reached, *p* = 0.004) [16]. Another current randomizing phase III study is the multicenter COMPETE trial, which aims to compare survival in GEP-NET (gastro-entero-pancreatic neuroendocrine tumors) patients after PRRT and Everolimus (COMPETE study, ClinicalTrials.gov Identifier (CI): NCT03049189). The principle of molecular targeting of NETs with PRRT has also been applied to other cancer entities, for example in the treatment of prostate cancer with the prostate-specific membrane antigen radioligand PSMA-RLD [17,18,19,20].

Recent approaches to the management of NETs aim to enhance the effectiveness of PRRT. Some examples are SSTR-targeting with antagonists instead of agonists, PRRT with alpha particles instead of beta particles, intra-arterial instead of intravenous application of PRRT or the combination of PRRT with other treatments. Furthermore, there have been several attempts to improve the detectability of NET lesions in PET imaging and advances in pretreatment planning using dosimetry. Especially notable is PET imaging with radioligand Copper-64. Increasing interest is also being paid to the theranostics of SSTR-negative NETs, for example targeting the CTXR-4-receptor in purely differentiated NETs [21]. Table 1 outlines important features of the above-mentioned radionuclides and other radionuclides utilized in modern nuclear medicine.

This review will present promising new agents in the molecular imaging and therapy of NETs, novel combination therapies and new applications of existing molecular imaging modalities in nuclear medicine.

## 2. Imaging and Therapy of SSTR-Positive NETs

In this part, we describe first advances in existing imaging and therapeutic techniques, including a combination of imaging modalities and standardization of the imaging interpretation in SSTR-positive NETs. Second, we introduce new approaches in combined treatment. Finally, we present promising attempts to increase tumor tracer retention and subsequent tumor detectability in the imaging and, more importantly, to increase the tumor dose for the therapy.

### 2.1. Refining Established Molecular Imaging and Treatment Regimens

In several studies, SSTR imaging was shown not only to correlate with the gene expression of SSTR in NETs but also with the Ki-67-index, and it was found to be an important predictive factor for survival [23,24,25,26,27]. Furthermore, the molecular imaging of NETs has a major impact on treatment planning [28]. However, there was a lack of standardization in the interpretations of the findings. Werner et al. introduced the first version of a structured reporting system for SSTR PET imaging on a 5-point scale: benign (1), likely benign (2), intermediate (3), highly likely NET (4) and almost certainly NET (5). The so-called SSTR-RAD score can be applied for the standardized assessment of diagnosis and might support treatment planning for patient selection for PRRT [29].

Most centers perform 3–5 cycles of PRRT; however, the current guidelines have no strict treatment scheme [30]. Some patients respond to initial PRRT but eventually relapse. The Bonn study group showed in selected patients that even 13 cycles of PRRT with [^177^Lu]Lu-DOTA-TATE and cumulatively administered activity up to 95.6 GBq is well tolerated with mostly mild/moderate toxicity [31,32,33]. Furthermore, several recent studies have shown that salvage PRRT is safe and that prolonged overall survival of 71–105 months can be reached [33,34,35,36].

### 2.2. Combined Imaging SSTR-PET and [^18^F]F-FDG-PET and Combined Treatment PRRT and Chemotherapy

In recent years, SSTR imaging and [^18^F]F-Fluorodeoxyglucose (FDG)-PET have not been considered as competitive but as complementary imaging tools able to help with choosing individualized treatments [37]. The scoring system (NETPET grade) of Chan et al. divides findings according to tumor uptake in SSTR-PET and [^18^F]F-FDG-PET: both SSTR-negative and FDG-negative, SSTR-positive and FDG-negative, and both SSTR- and FDG-positive (Table 2). Compared with the initial World Health Organization (WHO) tumor grade in the study, the NETPET grade shows significant correlation with OS (*p* not significant vs. 0.0018) [38]. Another study confirmed the correlation of SSTR- and FDG-PET with the cell differentiation of the tumor and highlighted that combined imaging is especially suitable for patients with Ki67 > 10% [39]. Combined imaging can help to select patients with high-grade NETs who are likely to benefit from a combined treatment of PRRT and chemotherapy (PRCRT) with comparable results to G1/G2 tumors [40,41]. In fact, in nearly 60% of NET patients, combined imaging had an impact on the therapeutic decision [42].

The NORDIC NEC study evaluated 305 patients with G3-neuroendocrine cancer (NEC) and revealed that NEC patients with Ki67 < 55% were less responsive to chemotherapy than patients with Ki67 > 55% [43]. In a retrospective analysis, patients with neuroendocrine neoplasia (NEN) Ki67 < 55% treated with PRCRT reached a median OS of 46 months, which was significantly longer than the reported OS of 14 months in the NORDIC trial with chemotherapy alone [43,44]. Other recent retrospective analyses with relatively heterogeneous patient cohorts showed disease control in up to 55–70% of patients [45,46]. The clinical benefit rates in these patients were not as high as the previously published results from the phase I/II studies by the Melbourne study group: 91–94% [47,48]. However, in such an extensively pre-treated cohort of patients with multiple relapses, PRCRT seems to be a good option. On the other hand, after a median follow-up of 32 months, the Australasian phase II study showed that PRCRT in PRRT-naïve patients did not result in longer PFS compared to patients who received PRRT alone. Furthermore, there was more toxicity in the PRCRT-group. These preliminary results question the benefit of combined treatment in PRRT-naïve patients and further phase III evaluation. Thus, longer follow-up is needed to determine if the efficiency of PRCRT is superior compared to PRRT alone [49]. Further prospective trials evaluating the combination treatment are expected be completed at the end of 2020 (CI: NCT02736448; NCT02358356).

Another promising combination in the future might be PRRT and small-molecule poly (ADP-ribose) polymerase-1 (PARP) inhibitors such as talazoparib. In a preclinical study, the combined treatment led to increased DNA double strand breaks compared to PRRT alone and improved the anti-tumor efficacy of PRRT, resulting in significantly longer survival. These findings support the clinical evaluation of combination therapy in NET patients [50].

### 2.3. The Status of PET/MRI in NETs

There is limited data about the advantages of PET/MRI over PET/CT. On the one hand are the higher costs and complexity of conducting these markedly time-consuming studies and interpreting the findings. On the other hand, PET/MRI could reduce radiation exposure, increase soft tissue contrast and conduct multiparametric evaluation, allowing better comprehension of tumor biology [51]. In NETs, there seems to be a synergistic benefit of PET/MRI in the detection of liver metastases, which are most common in metastatic abdominal NETs and are prognostic for survival. Limitations of the MRI are, first, the detection of pulmonary lesions and, second, the identification of hypersclerotic bone lesions in comparison to other imaging modalities [52].

Sawicki et al. reported in a head-to-head comparison of [^68^Ga]Ga-DOTA-TOC PET/MRI and [^68^Ga]Ga-DOTATOC PET/CT, that PET/MRI identified more NET lesions and provided better lesion conspicuity than PET/CT. The standardized uptake values (SUV) of the two modalities showed a strong correlation and no significant difference [53]. To date, PET/MRI has limited availability, and protocols and indications are still in development. For that reason, it is unlikely that PET/MRI will replace PET/CT in the near future [51].

### 2.4. SSTR-PET with Copper-64

Of several copper radionuclides that are potentially useful in medicine, Copper-64 is currently the most studied and well-known copper radioisotope. It is a promising all-round isotope with a convenient half-life for imaging and dosimetry studies, a combined spectrum of emitted radiation (β+, β−, γ) and formation of stable complexes with chelating molecules. Copper-64 can be produced in a reactor or a cyclotron [54,55].

A study group from Copenhagen, Denmark, compared the imaging characteristics of [^64^Cu]Cu-DOTA-TATE PET/CT with the most widely used PET tracer in NET [^68^Ga]Ga-DOTATOC PET/CT. The PET/CT with Copper-64 revealed more additional tumor lesions (*p* = 0.013) and fewer false-positives (1/42 vs. 18/26) than imaging with Gallium-68. One reason might be the relatively low positron range of Copper-64, which leads to an excellent spatial resolution. Another advantage of Copper-64 for clinical imaging is its physical half-life of 12.7 h, which allows late imaging and thus more scheduling flexibility in cases when patients show up late. One concern of [^64^Cu]Cu-DOTA-TATE PET/CT might be the higher radiation burden: the injection of 180–220 MBq per patient corresponds to a radiation dose of 5.7–8.9 mSv, which is approximately two times higher than after a standard dose of [^68^Ga]Ga-DOTA-TOC (120–200 MBq, 2.8–4.6 mSv radiation dose) [56]. The subsequent report about 500 patients from Copenhagen confirmed the safety and excellent diagnostic performance of [^64^Cu]Cu-DOTA-TATE. In a sub-analysis, the determined sensitivity was 97% (CI: 91–99%) and the specificity was 100% (CI: 96–100%) [57]. Additionally, a prospective, reader-blinded imaging trial with [^64^Cu]Cu-DOTA-TATE PET/CT from the USA provided outstanding results with 100.0% sensitivity, 97% specificity, accurate distinguishability between localized and metastatic tumors and excellent inter- and intra-reader reliability [58]. Recent data show that SSTR-PET/CT with Copper-64 with a SUVmax cutoff of 43.3 could predict PFS after 24 months of follow-up with a moderate accuracy of 57%. However, a matched comparison with the predictive value of Gallium-68-labelled imaging in the same population was missing in the study [26].

One disadvantage of imaging with [^64^Cu]Cu-DOTA-TATE PET/CT is the poor lesion uptake of the radiopharmaceutical at late time points. The reason might be that DOTA complexes with copper are very unstable under acidic conditions, and thus DOTA is not optimal for Cu-64 chelating [59]. In preclinical tests, the new agent [^64^Cu]Cu-SARTATE from Australia showed high stability in vitro and in vivo and seems to be a promising candidate to take advantage of the long half-life of Copper-64 and to enable late time imaging. In a small pilot trial, [^64^Cu]Cu-SARTATE achieved high tracer uptake and retention in lesions both at early time points (30 min and 1 h after administration) and late time points (4 h and 24 h after administration) with comparable or superior lesion detection to [^68^Ga]Ga-DOTATATE PET/CT. Especially in the liver, there was a significantly better detectability with [^64^Cu]Cu-SARTATE because of the clearance in the liver. Thus, [^64^Cu]Cu-SARTATE can be applied for diagnostic and for dosimetry studies for PRRT as well [60].

Another promising radiopharmaceutical that is still in preclinical testing is [^64^Cu]Cu-NODAGA-JR11, a somatostatin receptor 2 antagonist, which showed superior pharmacokinetic properties compared to [^64^Cu]Cu-DOTA-TATE, especially regarding tracer retention at late time points. These results endorse further clinical testing [61].

Significantly improved imaging properties with better contrast and detectability in a mice model in comparison to [^68^Ga]Ga-DOTA-TATE and [^64^Cu]Cu-DOTATATE could be reached with Cobalt-55-labelled DOTA-TATE. Furthermore, dosimetry calculations showed comparable effective doses between the three PET-tracers. Translation into clinical testing of [^55^Co]Co-DOTA-TATE is warranted [62].

### 2.5. PRRT and Liver Radioembolization

In recent analyses of the NETTER-1-study, Strosberg et al. postulated that NETs with large SSTR-positive target lesions (>3 cm diameter) had significantly shorter PFS than patients with small lesions (*p* = 0.022) [63]. Lutetium-177 (Lu-177) has a maximum tissue penetration of only 3 mm, which might be one reason for the worsened PFS of patients with large metastases. Another reason might be that most of the patients with large lesions had large liver lesions (70%), and high liver burden is associated with worse prognoses [64]. Such patients might need combined treatment with Yttrium-90 (Y-90) PRRT or liver-targeted treatment, such as Y-90 or Holmium-166 (Ho-166) radioembolization. Recent data from Braat et al. show that radioembolization of liver metastases is feasible in NET after initial PRRT. The objective responses after Y-90 and Ho-166 radioembolization were 16% and 43%, respectively. Each study included one patient with fatal radioembolization-induced liver disease [65,66]. Thus, more data about combined PRRT and liver radioembolization, especially regarding liver toxicity, are needed. Furthermore, in the abovementioned studies by Braat et al., it was not mentioned if the liver metastases after PRRT were still SSTR-positive [65,66]. However, this is important because selected patients who relapse after PRRT but still have adequate SSTR-expression of the target lesions are likely to respond to salvage PRRT [31,33]. Conversely, dedifferentiation of the NET lesions and subsequent loss of the SSTR expression has been associated with poor treatment response and short survival [29]. For these patients, effective therapies are urgently needed. Combination therapies for heterogeneous tumor lesions might be an optimal solution.

### 2.6. Small Change, Big Difference: Evans Blue Modification of DOTA-TATE to Increase Tumor Retention

PRRT with [^177^Lu]Lu-DOTA-TATE is a safe treatment that prolongs the survival of NET patients; however, the response rate is relatively low (18%) [16]. To overcome this problem, modifications of the radionuclide or peptide might be necessary.

A study group from Bethesda, Maryland (US) conjugated DOTA-TATE with an Evans blue analog (EB), which irreversibly binds to serum albumin. This modification should lower renal clearance and increase blood circulation half-life, resulting in higher accumulation in SSTR-positive tumors. In mouse models, the radiolabeled conjugate DOTA-EB-TATE with Yttrium-90 showed significantly better tumor response and survival compared to radiolabeled DOTA-TATE (*p* < 0.001) [67]. A prospective pilot study evaluating the effect of [^177^Lu]Lu-DOTA-EB-TATE compared to [^177^Lu]Lu-DOTA-TATE in patients with advanced NETs was conducted soon after these promising results. Four patients were included in the [^177^Lu]Lu-DOTA-EB-TATE group and three patients were included in the [^177^Lu]Lu-DOTA-TATE group. The head-to-head study showed comparable results in both treatment groups even after low-dose treatment of [^177^Lu]Lu-DOTA-EB-TATE (approximately 1/6 of the DOTA-TATE dose). With [^177^Lu]-DOTA-EB-TATE, a 7.9-fold increase in the tumor dose delivery could be reached. There were no significant adverse events. Further studies with higher activities and more cycles with [^177^Lu]Lu-DOTA-EB-TATE should follow [68].

### 2.7. PRRT with Antagonists

The internalization of SSTR agonists together with the receptor in tumor cells allows direct tumor irradiation, which was supposed to be an advantage for agonists until Ginj et al. showed in 2006 that SSTR-antagonists have superior tumor uptake because of increased binding sites at the receptor and a lower dissociation rate [69].

Meanwhile, there have been several preclinical and clinical studies that investigated different radiolabeled SSTR antagonists and demonstrated their superior tumor uptake compared to SSTR agonists, along with an excellent tumor-to-kidney-dose ratio [70,71,72,73]. In a recent head-to-head comparison of the SSTR agonist [^68^Ga]Ga-DOTA-TATE and the SSTR-antagonist [^68^Ga]-DOTA-JR11, in the PET/CT with the antagonist, more liver metastases could be detected, but the agonist was superior in the detection of osseous metastases. These findings might be relevant for choosing the appropriate imaging and peptide receptor radionuclide therapy according to the metastatic spread of the tumor [74].

The most-studied SSTR-antagonist-radiopharmaceuticals to date are [^68^Ga]Ga-NODAGA-JR11 and [^68^Ga]Ga-DOTA-JR11 for PET imaging and [^177^Lu]Lu-DOTA-JR11 for treatment [75] (CI: NCT02609737). The newest data confirm the largely concordant uptake from [^68^Ga]Ga-DOTA-JR11-PET and post-[^177^Lu]Lu-DOTA-JR11-SPECT/CT, making [^68^Ga]Ga-DOTA-JR11 and [^177^Lu]Lu-DOTA-JR11 a suitable theranostic pair [76]. Still, there is a demand for comparative studies between SSTR agonists and SSTR antagonists in the treatment of NETs.

### 2.8. Alpha Radiopeptide Therapy

Alpha particles cause more double strand DNA breaks than beta particles because of the formers’ much higher linear energy transfer. Furthermore, the short range of alpha particles preserves the surrounding non-tumor tissue. Targeted alpha therapy can be applied to enhance the anti-tumor-effect and to overcome beta radiation resistance in SSTR-positive NETs after PRRT with beta particles [77]. A recent clinical report about alpha-PRRT with [^225^Ac]Ac-DOTA-TATE in relapsed NETs after beta PRRT revealed a surprisingly high response: 63% objective response and 100% clinical benefit rate. No toxicity was observed [78]. A positive experience with alpha PRRT ([^255^Ac]Ac-DOTATOC) was also reported by a study group in Bad Berka, Germany [79]. A newly tested radiopharmaceutical for targeted alpha therapy in NETs is [^212^Pb]Pb-DOTAMTATE (AlphaMedix™). Lead-212 is a beta particle and the parent nuclide of the alpha particle Bismuth-212; in this way it acts as an “in vivo generator” for alpha particles. The study objectives are the assessment of the safety and efficacy of [^212^Pb]Pb-DOTAMTATE. The study is currently recruiting and should include 50 “PRRT-naïve” patients with SSTR-positive NETs (CI: NCT03590119).

## 3. Imaging and Therapy of SSTR-Negative NETs

The management of SSTR-negative NETs is challenging. The development of better diagnostic and therapeutic modalities is increasingly important. This chapter summarizes novel targets in well differentiated or purely differentiated SSTR-negative NETs. Table 3 illustrates different radiopharmaceuticals used in molecular imaging and treatment.

### 3.1. CXCR4 Targeting for Imaging and Treatment

Chemokines accelerate malignant transformation and metastatic spread [95]. Several mostly preclinical studies have reported about C-X-C motif chemokine receptor 4 (CXCR4)-targeted molecular imaging and therapy [29,54,82,83,96,97,98,99,100,101,102]. CXCR4 is overexpressed in GEP-NETs and can act as a potential target for treatment.

Werner et al. conducted a comparison between the PET tracers [^68^Ga]Ga-Pentixafor, [^68^Ga]Ga-DOTA-TOC and [^18^F]F-FDG in twelve patients with GEP-NETs (G1-G3). Pentixafor (approved by the FDA in 2008) is an analog of the CXCR4-ligand chemokine ligand 12/stromal cell-derived factor-1 (SDF-1) [103,104]. The study showed that patients with high-grade tumors exhibited [^68^Ga]Ga-Pentixafor-positive tumor lesions: 50% in G2 and 80% in G3 tumors. Thus, CXCR4 targeting for imaging is a promising option for patients with advanced dedifferentiated SSTR-negative tumors [29]. However, because of its relatively fast clearance rate, [^68^Ga]Ga-Pentixafor might not be suitable for treatment. The novel agent Pentixather shows considerable stability and a similar affinity to CXCR4 as that of its analog Pentixafor [81]. The first clinical experiences with Lutetium-177 and Yttrium-90 labelled Pentixather as combined treatment with chemotherapy and autologous stem cell transplantation showed a very good response rate in hematological diseases: two of three patients with advanced multiple myeloma [82] and four of four patients (two partial response, two mixed response) in diffuse large B-cell lymphoma [84].

Although the study only addressed a limited number of treated patients, the high response rate in such heavily pretreated patients and the good toxicity profile indicates that endoradiotherapy targeting CXCR4 might be the next breakthrough in the field of nuclear theranostics. Future studies should test the effectiveness of this treatment in selected NET patients.

### 3.2. FAPI Imaging in NETs

Cancer-associated fibroblasts overexpress, in contrast to normal fibroblasts, the fibroblast activation protein (FAP), which contributes to tumor growth and metastatic spread in several tumor entities [105]. Therefore, radiolabeled, FAP-specific inhibitors might be promising tumor-targeting radiopharmaceuticals in NETs [106,107].

In 2019, Kratochwil et al. published a study with [^68^Ga]Ga-FAPI PET in 28 different kinds of cancer, including NET. The three included cases of NETs showed intermediate tracer uptake with tumor-to-background ratios higher than three. These results were surprising because, in the histopathology, NETs did not show such high desmoplastic reactions as tumor entities such as colorectal and pancreatic cancer, but the tumor uptake in the imaging was similar to these and other cancer types such as head-neck, hepatocellular, ovarian and prostate cancers [85]. Thus, in NETs with disseminated metastatic disease, FAPI imaging can contribute to noninvasive tumor characterization and the individualization of treatment planning.

### 3.3. GLP-1 Receptor Radiopharmaceuticals

Insulinomas are mostly benign tumors; however, the upregulated insulin secretion can be difficult to manage and even life threatening. A vital point for the effective treatment of insulinomas in localized tumors with surgical resection is the reliable detection of the typically very small tumor. A useful but invasive technique in this setting is arterial, calcium-stimulated venous sampling. Insulinoma cells have high glucagon-like peptide-1 (GLP-1) receptor expression in contrast to SSTR expression (sensitivity of SSTR-PET about 25%). Exendin is a ligand for the GLP-1 receptor and, as a radiolabeled substance, can be applied in the imaging of insulinomas. Several recent studies have shown excellent results with this technique, even in cases in which CT/MRI imaging has failed [86,88]. In a prospective cohort study, Luo et al. tested [^68^Ga]Ga-NOTA-Exendin-4 in the detection of localized insulinomas and provided excellent results: 98% sensitivity and SUVmax 23.6 +/−11.7. In comparison, MRI had a sensitivity of 56% and [^99m^Tc]Tc-hydrazinonicotinamide (HYNIC)-octreotide only 20% [87]. Thus, more prospective studies and experiences in the clinic setting are needed to validate these data.

### 3.4. CCK2R Targeting in SSTR-Negative NETs

The cholecystokinin 2 receptor (CCK2R) is a G-protein coupled transmembrane receptor, which is highly overexpressed in several neuroendocrine tumor types, such as medullary thyroid cancer (MTC), stomach NETs, pancreatic NETs and bronchopulmonary NETs [91]. For example, 91–100% of MTCs show a high density of CCK2R [91,108]. However, although CCK2R has been known as a promising theranostic target for about two decades, the clinical data to date have been very limited. Specifically, nephrotoxicity was the main limiting factor for the therapeutic application of the previously tested Yttrium-90-labelled minigastrin in MTC [108].

A novel agent [^177^Lu]Lu-DOTA-(d-Glu)6-Ala-Tyr-Gly-Trp-Nle-Asp-PheNH2 ([^177^Lu]Lu-PP-F11N) has been tested in a pilot randomized prospective trial (ClinicalTrials.gov: NCT02088645) and has shown very promising results with high tumor uptake and low kidney/bone marrow uptake. However, the stomach uptake was relatively high (tumor-to-stomach dose ratio of 3:34), and for that reason the stomach seems to be most likely a dose-limiting organ. Apart from that, the adverse events, such as flushing, hypokalaemia and hypotension were mild and self-limiting. Thus, [^177^Lu]Lu-PP-F11N seems to be a very promising treatment option in patients with advanced metastatic MTC or as adjuvant treatment in high-risk patients after they have been operated on [90]. Furthermore, [^177^Lu]Lu-PP-F11N should be tested in SSTR-negative, CCK2R-positive NETs. Reliable imaging with radiolabelled minigastrin analogues is required to select suitable patients. One promising PET tracer is [^68^Ga]Ga-DOTA-PP-F11 [92]. However, more clinical data about CCK2R-targeting theranostics are needed.

## 4. Current Phase III Trials

There are several ongoing phase III trials for molecular imaging and treatment in NET. The COMPETE trial (CI: NCT03049189) is a multicenter, prospective study that plans to enroll 300 patients with advanced, progressive SSTR-positive GEP-NET, who either receive PRRT (4 cycles) or the mammalian target of rapamycin (mTOR) inhibitor Everolimus (10 mg/d until progression or end of the study). The aim of the study is to compare the toxicity and efficacy of PRRT with Everolimus. The estimated study completion date is May 2024.

Another current study is evaluating the predictive role of different imaging methods: PET with [^68^Ga]Ga-DOTA-TATE and [^18^F]F-FDG, diffusion weighted MRI and post-[^177^Lu]Lu-DOTA-TATE SPECT/CT dosimetry in tumor response after PRRT (CI: NCT01842165).

A multicenter randomized controlled trial from the Netherlands (phase II/III) aims to compare intravenous and intra-arterial PRRT administration. Patients with metastases in both liver lobes will receive intra-arterial-PRRT (first-pass effect) in one of the liver lobes, while the contralateral lobe will receive intravenous PRRT (second-pass effect). The study should be completed by September 2021 (CI: NCT03590119).

As several important trials are on-going and further crucial clinical studies need to be carried out, an update will be needed when the trials are complete.

## 5. Summary

The management of NETs remains challenging. Different tumor-specific biological indicators in NETs have been developed in molecular imaging to show their activity in vivo and post-therapeutic changes over time. New advances in nuclear medicine promise to enhance the detectability of NETs in molecular imaging and to provide a non-invasive characterization of tumor biology. Further attempts are needed to increase the efficacy of PRRT to achieve more objective responses and longer PFS. Novel molecular therapies and combination treatments should improve the outcome of fast-growing, multiple relapsing and purely differentiated NETs. Finally, more prospective randomized trials with large numbers of patients should validate the value of nuclear medicine theranostics in NETs.

## Figures and Tables

**Table 1:** Diagnostic and therapeutic radionuclides.

Radionuclide	Emitted Particle	Half-Life	Utilization
Carbon-11	beta blus	20 min	Imaging
Cobalt-55	beta plus	17.5 h	Imaging
Copper-64	beta plus beta minus	12.7 h	Imaging Therapy
Gallium-68	beta plus	68 min	Imaging
Fluorine-18	beta plus	120 min	Imaging
Actinium-225	alpha decay	10 d	Therapy
Bismuth 212	alpha decay (36%) beta minus (64%)	60.6 min	Therapy
Bismuth-213	alpha decay beta minus gamma coemission	46 min	Therapy
Holmium-166	beta minus	1.1 d	Therapy
Lead-212	beta minus	10.6 h	Therapy
Lutetium-177	beta minus gamma	6.7 d	Therapy
Yttrium-90	beta minus	2.7 h	Therapy

Reference: Blower et al. [22].

**Table 2:** The NETPET scores with a relevance of the peptide receptor radionuclide therapy (PRRT) indication.

Score PET	P0	P1	P2	P3	P4	P5
SSTR	-	+	+	+	+	-
FDG	-	-	+	+	+	+
Correlation SST/FDG-Uptake	not applicable	not applicable	SSTR > FDG	SSTR = FDG	SSTR < FDG	-
Indication for PRRT	no	yes	yes	evtl. PRRT combined with chemotherapy	no	no

Reference: Chan et al. [38].

**Table 3:** Potential targets for molecular imaging and treatment in somatostatin receptors (SSTR)-negative neuroendocrine tumors (NET).

Tracer	Target	Application	Tumor Feature/ Tumor Type	Reference
[^18^F]F-FDG	Glucose transporter (Glut)-1	Diagnostic and prognostic	Hypermetabolic, rapidly proliferating tumors	[80]
[^68^Ga]Ga-Pentixafor/[^177^Lu]Lu/[^90^Y]Y-Pentixather	C-X-C motif chemokine receptor 4 (CXCR4)	Diagnostic and therapeutic	High grade NET	[29,81,82,83,84]
[^68^Ga]Ga-FAPI	Fibroblast activation protein (FAP)	Diagnostic	Cancer-associated fibroblasts in mainly high grade NET	[85]
[^68^Ga]Ga-NOTA-Exendin-4	Glucagon-like peptide-1 (GLP-1) receptor	Diagnostic	Insulinoma	[86,87,88,89]
[^68^Ga]Ga-DOTA-PP-F11/[^177^Lu]Lu-PP-F11N	Cholecystokinin 2 Receptor (CCK2R)	Diagnostic and therapeutic	Medullary thyroid cancer, stomach NET, pancreas NET and bronchopulmonary NET	[90,91,92]
[^18^F]F–DOPA	Catecholamine-producing chromaffin cells of NET	Diagnostic	Medullary thyroid cancer, midgut NET, pheochromocytoma, neuroblastoma, paraganglioma	[93]
[^123^I]I-/[^131^I]I-MIBG	Metaiodobenzylguanidine (MIBG) is similar to the neurotransmitter norepinephrine and enters neuroendocrine cells from the sympathetic nervous system	Diagnostic and therapeutic	Pheochromocytoma, neuroblastoma, paraganglioma	[94]
